# Prey preference and cell wall-mediated resistance shape predation efficiency in *Saccharomycopsis schoenii*

**DOI:** 10.1093/femsyr/foaf075

**Published:** 2026-01-02

**Authors:** Jan Ryno Smith, Rene K Naidoo-Blassoples, Florian F Bauer

**Affiliations:** South African Grape and Wine Research Institute, Department of Viticulture and Oenology, Stellenbosch University, JH Neethling building, Victoria Street, Private Bag X1, Matieland 7602, South Africa; South African Grape and Wine Research Institute, Department of Viticulture and Oenology, Stellenbosch University, JH Neethling building, Victoria Street, Private Bag X1, Matieland 7602, South Africa; South African Grape and Wine Research Institute, Department of Viticulture and Oenology, Stellenbosch University, JH Neethling building, Victoria Street, Private Bag X1, Matieland 7602, South Africa

**Keywords:** yeast predation, resistance, prey preference, killing efficiency, predator–prey interactions

## Abstract

Microbial antagonism, including predation and competition, shapes microbial community diversity and dynamics. *Saccharomycopsis schoenii*, a unicellular predatory yeast, serves as a distinct model for *bona fide* fungal predation, characterized by penetration pegs that enable predation. This study examined prey preferences of *S. schoenii* within wine-associated yeast consortia and assessed the role of prey adhesion and cell wall features in modulating predation efficiency. Predation assays revealed species-specific dynamics, with *Saccharomyces cerevisiae* showing pronounced susceptibility and *Torulaspora delbrueckii* displaying resistance indicative of density-dependent prey switching. Expression of prey Flo-adhesins in *S. cerevisiae* did not affect predation outcomes, highlighting that prey adhesion phenotypes are not primary determinants of susceptibility. In contrast, *S. cerevisiae* VIN13-related mutant strains with increased cell wall chitin showed variable resistance phenotypes, suggesting that chitin contributes to resistance, but that broader cell wall remodelling and structural features are relevant factors independent of chitin levels. While these findings provide a mechanistic framework for understanding predator–prey interactions and prey resistance, the ecological and evolutionary significance of these interactions remains uncertain due to the rarity of *Saccharomycopsis* species in natural communities. Ultimately, these results emphasize the importance of integrating laboratory and ecological perspectives to fully comprehend the evolutionary implications of fungal predatory behaviour.

## Teaser

The predation efficiency of *Saccharomycopsis schoenii* against selected wine yeast species as prey, as well as the impact on predation efficiency of prey cell wall-related phenotypes, was investigated.

## Introduction

Microbial antagonism primarily involves key ecological interactions such as amensalism, competition, parasitism, and predation. These antagonistic interactions are essential in shaping the diversity and dynamics of microbial communities (Feichtmayer et al. 2017, Giometto et al. 2021). In fungi, filamentous species use toxin and enzyme gradients to suppress competitors or parasitize other fungi (Jeffries 1995, Hibbing et al. 2010). These competitive strategies define niche boundaries and form complex networks of competition. For instance, in fungal communities, toxin production and mycoparasitism restrict overlapping growth, resulting in certain fungi persisting in specific microhabitats, while others are excluded, thus shaping unique niches and enabling coexistence (Crowther et al. 2014). Yeasts, often considered less aggressive antagonists, employ sophisticated attack and defence mechanisms, including the secretion of proteinaceous 'killer toxins' that support competition, especially in mixed-species environments (Starmer et al. 1987, Schmitt and Breinig 2006, Boynton 2019, Giometto et al. 2021). The discovery of yeasts in the *Saccharomycopsis* genus (formerly *Arthroascus*), a unique group of unicellular predators that directly penetrate and kill other yeasts, has broadened our understanding of yeast–yeast antagonism into a new realm (Lachance and Pang 1997, Lachance et al. 2000, Kurtzman and Smith 2011, Junker et al. 2019, Santos et al. 2023).

Species belonging to the genus *Saccharomycopsis* are the best-characterized examples of predatory yeasts following a *bona fide* predatory lifestyle. Members of this genus are unable to assimilate inorganic sulphate and instead rely on organic sulphur sources such as methionine (Lachance et al. 2000, Junker et al. 2019). Under conditions of nutrient stress, especially sulphur limitation, *Saccharomycopsis schoenii* and related species initiate predation by producing specialized infection structures, 'penetration pegs', that physically breach the cell wall of a prey cell (Lachance and Pang 1997, Junker et al. 2018, Rij et al. 2024). Following penetration, the predator extracts nutrients from its prey, leading to cell death. Previous work has demonstrated that *S. schoenii* efficiently kills multidrug-resistant *Candidozyma auris* (formerly *Candida auris*) strains and can be utilized effectively in post-harvest agricultural practices and even in the production of aged meat products (Pimenta et al. 2008, Iacumin et al. 2017, Junker et al. 2018). This highlights both its ecological breadth and its potential as a biocontrol agent in clinical and agricultural settings.

Recent studies have partially elucidated the predatory mechanisms of *S. schoenii* (Junker et al. 2019, Rij et al. 2024). They demonstrated that *S. schoenii* upregulates proteases, glucanases, and chitinases when it encounters prey. Rij et al. (2024) also identified gene families and signalling pathways involved in controlling this response. The resulting enzymes act to degrade prey cell wall polysaccharides, which are essential for maintaining cell integrity. However, a comprehensive understanding of the ecological, physiological, and molecular aspects of both the predatory process and its function within its natural niche is still lacking. For example, research on *Saccharomycopsis fibuligera* employs diverse approaches to characterize its role in food fermentation, yielding insights that go beyond predatory enzyme profiling (Methner et al. 2022). Important unanswered questions remain regarding the natural ecological niche of *Saccharomycopsis* yeasts, the significance of predation within these systems, and the physiological, cellular, and molecular mechanisms underpinning their predatory behaviour.

*Saccharomycopsis* species have been isolated from oak exudate, rotten apples, and soil in apple orchards, as well as from plant material in managed settings such as botanical gardens, vineyards, papaya plantations, and insects, suggesting relatively wide distribution (Kurtzman and Smith 2011, Lachance et al. 2012, Santos et al. 2023, Dost et al. 2024, Hu et al. 2025). This is, however, mitigated by the fact that there are relatively few data showing a significant presence of this genus in these same ecosystems. Furthermore, there are currently no quantitative data describing co-occurring yeast species within natural habitats, and consequently, no information available on ecologically and evolutionarily relevant prey. Even in the few quantitative studies available from anthropic environments, such as Mizuno et al. (2024) who reported *S. fibuligera* at 2.5% relative abundance as a wild contaminant in industrial soy sauce fermentation, the ecological context differs substantially from natural habitats, where predation may play evolutionary roles. The predation process itself involves several steps, beginning with adhesion of the predator to the prey, formation of the penetration peg, digestion of prey cell walls, and finally the absorption of relevant organic molecules. While some of these steps have been partially characterized, there is no data on the molecular responses of prey or the mechanisms of prey resistance to predation (Junker et al. 2018, 2019, Rij et al. 2024).

Despite a lack of evidence tying *Saccharomycopsis* yeasts to wine fermentation environments, wine yeast consortia provide an experimentally tractable model system for investigating predatory yeast interactions. Fermentation environments support complex communities comprising *Saccharomyces cerevisiae* and diverse non-*Saccharomyces* species, including *Torulaspora delbrueckii* and *Lachancea thermotolerans*, among others, which exhibit well-characterized ecological interactions (Bagheri et al. 2020, Vaquero et al. 2021, Conacher et al. 2022, Pourcelot et al. 2025). *Saccharomyces cerevisiae*, as a model organism, offers experimental advantages in phenotypic diversity in adhesion properties and cell wall composition (Lesage and Bussey 2006, Brückner and Mösch 2012, Rossouw et al. 2018). Furthermore, the reasonably well-characterized cell wall architecture of *S. cerevisiae*, combined with a variation in Flo-adhesins, allows for a systematic analysis of surface properties in modulating predation efficiency (Klis et al. 2002, Lesage and Bussey 2006, Willaert 2018). Taken together, the use of wine-related *S. cerevisiae* strains offers a promising candidate prey species for investigating prey preferences and killing efficiency of *S. schoenii*.

This study screened *S. schoenii* prey preferences for several species that are prominent within the wine yeast fermentation ecosystem. Specific prey preferences were detected among yeasts associated with fermentation ecosystems. *Saccharomyces cerevisiae* wine yeast strains were used to assess the impact of prey adhesion phenotypes and the cell wall composition on predation efficiency.

## Methods and materials

### Yeast strains and preculture conditions

All yeasts used in this study are listed in Table 1. All strains were maintained at −80°C in 15% glycerol solution. The strains HCVin-1 to -5 are derivatives of the *S. cerevisiae* wine yeast VIN13 with increased chitin levels that were generated by ethyl methanesulfonate (EMS) mutagenesis as described by Chuene et al. (2024). Yeast strains were streaked from cryo-stocks on Wallerstein Laboratory (WL) nutrient agar (Merck, Darmstadt, Germany) plates and incubated at 30°C for 2 days before preculturing.

**Table 1.** Yeast strains used in this study.

Yeast	Strain/Mutant	Genotype	Reference
*Saccharomycopsis schoenii*	CBS 7425	Wildtype	Westerdijk Fungal Biodiversity Institute (Utrecht, The Netherlands)
*Torulaspora delbrueckii*	LO544	Wildtype	Centre de Ressources Biologiques Occitanie (France)
*Lachancea thermotolerans*	CBS 16 374	Wildtype	Westerdijk Fungal Biodiversity Institute (Utrecht, The Netherlands)
*Saccharomyces cerevisiae*	VIN13	Commercial wine yeast strain (Wildtype)	Anchor Yeast (Cape Town, South Africa)
*Saccharomyces cerevisiae*	VIN13-F5H	FLO5p::SMR1-HSP30p	(Govender et al. 2010)
*Saccharomyces cerevisiae*	VIN13-F11H	FLO11p::SMR1-HSP30p	(Govender et al. 2010)
*Saccharomyces cerevisiae*	BM45	Commercial wine yeast strain (Wildtype)	Lallemand Inc. (Montreal, Canada)
*Saccharomyces cerevisiae*	BM45-F1H	FLO1p::SMR1-HSP30p	(Govender et al. 2010)
*Saccharomyces cerevisiae*	BM45-F5H	FLO5p::SMR1-HSP30p	(Govender et al. 2010)
*Saccharomyces cerevisiae*	BY4742	MATα his3Δ1 leu2Δ0 lys2Δ0 ura3Δ0	(Baker Brachmann et al. 1998; Euroscarf, Y10000)
*Saccharomyces cerevisiae*	BY4742 ΔFLO1	flo1Δ::KanMX4	(Baker Brachmann et al. 1998; Euroscarf, Y10000)
*Saccharomyces cerevisiae*	BY4742 ΔFLO5	Flo5Δ::KanMX4	(Baker Brachmann et al. 1998; Euroscarf, Y10000)
*Saccharomyces cerevisiae*	BY4742 ΔFLO11	flo11Δ::KanMX4	(Baker Brachmann et al. 1998; Euroscarf, Y10000)
*Saccharomyces cerevisiae*	HCVin-1	High Chitin Mutant (EMS-mutagenized) genotype unknown	(Chuene 2025)
*Saccharomyces cerevisiae*	HCVin-2	High Chitin Mutant (EMS-mutagenized) genotype unknown	(Chuene 2025)
*Saccharomyces cerevisiae*	HCVin-3	High Chitin Mutant (EMS-mutagenized) genotype unknown	(Chuene 2025)
*Saccharomyces cerevisiae*	HCVin-4	High Chitin Mutant (EMS-mutagenized) genotype unknown	(Chuene 2025)
*Saccharomyces cerevisiae*	HCVin-5	High Chitin Mutant (EMS-mutagenized) genotype unknown	(Chuene 2025)

### Preculture and predation screening conditions

Single colonies of each yeast strain were inoculated into 10 ml yeast extract-peptone-dextrose (YPD) broth (Sigma–Aldrich, Johannesburg, South Africa) in test tubes and incubated at 30°C on a test tube wheel rotating at 50 rpm, until log phase. Cells were then harvested by centrifugation at 2000 g for 3 min and resuspended in Erlenmeyer flasks containing 50 ml YPD broth and incubated in a Being Shaking incubator (Being Technology Co., Ltd, Kunshan City, China) at 40 rpm at 30°C for 16 h. A second preculturing step involved harvesting cells as discussed previously, washing cells with a 0.9% saline solution and transferring the biomass to Erlenmeyer flasks containing 100 ml of Yeast Nitrogen Base (YNB) (Becton, Dickinson and Company, Le Pont de Calix, France) broth in Erlenmeyer flasks, consisting of 6.7 g l^−1^ YNB broth without amino acids, with 5 g l^−1^ ammonium sulphate and 20 g l^−1^ glucose supplemented with Complete Supplement Medium less Methionine (CSM-Met) (Formedium, UK) in a shaking incubator at 30°C for 12 h until mid-log phase. *Saccharomycopsis schoenii* was cultured for 16 h until mid-log phase.

To investigate the impact of prey adhesion phenotypes on predation dynamics, *S. cerevisiae* BY4742 FLO deletion mutants, BM45- and VIN13 FLO-overexpressing mutants were screened. FLO-deletion mutants were precultured as described above. FLO-overexpressing mutants were subjected to heat shock at 42°C for 30 min to induce the FLO-overexpression phenotypes as described by Govender et al. (2008). Control wild-type (WT) strains were also subjected to heat shock during prey adhesion screening.

The culture medium used for predation screening consisted of 6.7 g l^−1^ YNB broth without amino acids, with 5 g l^−1^ ammonium sulphate and 20 g l^−1^ glucose, supplemented with CSM-Met according to manufacturer instructions. Predator–prey screens were performed by inoculating equal ratios of *S. schoenii* and prey yeast in 6-well flat-bottom Cellstar^®^ plates (Greiner Bio-One, Kremsmünster, Austria) containing 5 ml culture medium as mentioned above and incubated on a digital microplate shaker (Thermo Fisher Scientific, MA, USA) at 300 rpm at 25°C. Yeast consortia were prepared by inoculating prey species at equal densities of ∼2.5 × 10^6^ cells ml^−1^ and *S. schoenii* at 5 × 10^6^ cells ml^−1^ in 6-well flat-bottom Cellstar^®^ plates containing 5 ml culture medium and incubated at 300 rpm at 25°C. Directly after inoculation, 200 μL samples were taken, followed by every 20 min thereafter for the first hour and then every 30 min thereafter for 3 h total, except when stated otherwise. Samples were diluted 10x in a 1x solution of phosphate-buffered saline (PBS) and 20 mM ethylenediaminetetraacetic acid (EDTA). The quantification of yeast cells and the determination of population ratios were performed using a benchtop flow cytometer (CytoFlex, Beckman Coulter, CA, USA).

### Predation screening on agar plates

Predation screens were conducted on WL- and YNB CSM-Met agar plates. Before co-culture spotting of predator and prey yeast, single colonies of each were inoculated separately into 10 ml YPD broth and grown overnight at 30°C on a test tube wheel rotating at 50 rpm, until log phase. Overnight cultures were centrifuged at 2000 g for 3 min and resuspended in sterile PBS (Invitrogen, MA, USA) to achieve equal cell densities of 10^6^ cells ml^−1^. Ten microlitres of *S. schoenii* and prey was spotted next to each other but not touching, with an ∼1 mm gap between both spots. Plates were incubated at 30°C for a maximum of 10 days. The growth of both predator and prey spots was visually inspected to compare the encroachment of *S. schoenii* towards prey.​ Morphologies were imaged using a standard camera at ∼100 mm from the plate.

### Confocal microscopy, image processing, segmentation, and fluorescence quantification

In preparation for confocal microscopy, plate colonies were resuspended in 10 ml of YPD broth in test tubes on a test tube wheel at 50 rpm for 14 h at 30 °C. Cells were harvested by centrifugation and resuspended in 10 ml fresh YPD broth and incubated for a further 6 h. Cells were harvested once again, washed twice with a 1x PBS solution and cell density adjusted to 5 × 10^6^ CFU ml^−1^. Cells were stained with calcofluor white and prepared for confocal microscopy according to the methods described by (de Groot et al. 2001).

All images were acquired using an Olympus FV4000 (Evident Scientific, Tokyo, Japan) laser-scanning confocal mounted on an IX83 inverted microscope with a UPLXAPO 40×/0.95 NA dry objective (WD 0.18 mm; *n* = 1.0). Multichannel z-stacks (1024 × 1024 pixels in XY, multiple z-sections; voxel size 0.155 × 0.155 × 0.44 µm) were collected sequentially using resonant scanning. Excitation used LD405 lasers with system transmissivity set to 0.7%. Pinhole size was automatically adjusted to 1 Airy unit. Z-plane stacks were collected with resonant scanning in line-sequential mode. Acquisition parameters, including series dimensions (XYCZT), laser power, detector settings, and LUTs, were kept constant throughout the dataset to ensure comparability.

Image processing and fluorescence quantification followed a workflow modified from (Ash et al. 2021). OIR files were imported into Fiji/ImageJ (version 1.54p) (Schindelin et al. 2012) using Bio-Formats Importer. DAPI channels were Z-projected by maximum intensity; background was reduced (rolling ball, 150 px), and median filtering (radius 2 px) was applied. Segmentation involved thresholding with dark-background mode, hole filling, watershed separation, and particle analysis (8–150 µm², circularity 0.15–1.00), with objects screened manually. Fluorescence per cell was measured based on mean pixel intensity in each region of interest (ROI), and summaries were normalized to cell count. Cell-specific fluorescence intensity was quantified on the cleaned projection image. The fluorescence intensity was calculated across all cells and conditions to provide a per-image chitin intensity estimate (a.u.).

### Modelling prey decline and statistical analyses

Prey mortality was quantified by fitting cell counts to an exponential decay model where prey abundance changes over time (Beauchamp et al. 2007). Curve fitting was performed in Python version 3.13.1 (Python Software Foundation) using the SciPy optimisation library, and parameter estimation was carried out by nonlinear least squares regression (Minter et al. 2011). Mean killing rates were calculated from the change in prey cell counts between successive sampling intervals, expressed relative to the time elapsed to provide a standardized measure of the rate of decline for prey.

All statistical analyses were performed using R, version 4.5-arm64. Statistically meaningful differences in mean chitin fluorescence and killing efficiency between prey strains were determined by one-way analysis of variance (ANOVA) followed by Dunnett’s one-sided tests with *P*-values adjusted for multiple comparisons. Significance thresholds were set at *P <* 0.05. All graphical data are presented as mean ± standard error of the mean (SEM).

## Results

### Investigating prey preferences of *S. schoenii* in wine-related consortia and the impact of prey adhesion phenotypes on predation efficiency

**Figure 1.**
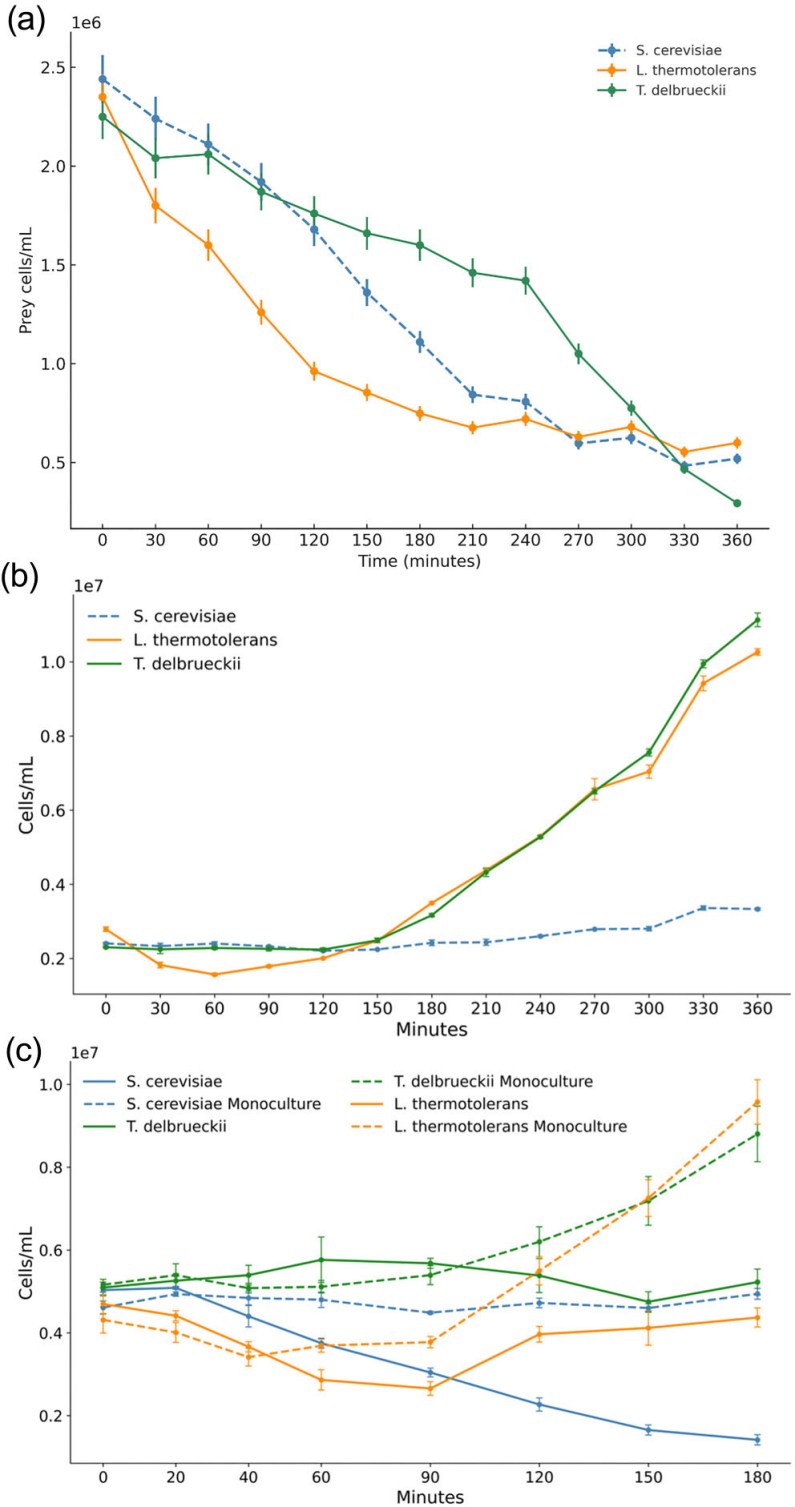
Investigating prey preferences between wine fermentation-related *Saccharomyces* and non-*Saccharomyces* yeast. (a) Line graph representing the mean prey cell density and decline for *S. cerevisiae* VIN13, *T. delbrueckii*, and *L. thermotolerans*, as prey, in a consortium together with *S. schoenii*. The plots show the decline in viable cell density over a 360-min assay period, reflecting trends of differential susceptibility of wine-related non-*Saccharomyces* yeasts compared to *S. cerevisiae* VIN13 control strain. (b) A three-species consortium of *S. cerevisiae* VIN13, *T. delbrueckii*, and *L. thermotolerans* with no *S. schoenii* present. (c) Comparative line graphs representing the mean prey cell density and decline for *S. cerevisiae* VIN13, *T. delbrueckii*, and *L. thermotolerans*, as prey, in co-culture with *S. schoenii*. The graphs in Fig. 1a and b represent yeast in a consortium. The graphs in Fig. 1c have been overlaid for comparison between species and monoculture and co-culture conditions with solid lines showing prey cell density in co-culture with *S. schoenii* and dashed lines showing monoculture growth for prey.

We screened a consortium consisting of *S. cerevisiae* VIN13, *L. thermotolerans, T. delbrueckii*, and *S. schoenii* to investigate whether *S. schoenii*, as the predator, shows specific prey preferences. The data in Fig. 1a indicate that all prey species declined from the earliest time point, albeit with slightly different rates of decline. *Torulaspora delbrueckii* initially exhibited a slower decline, while *L. thermotolerans* declined the fastest at first, and *S. cerevisiae* declined at an intermediate, linear rate. Once the concentrations of prey cells of *L. thermotolerans* and *S. cerevisiae* had stabilized at a low level, the decline of *T. delbrueckii* accelerated and reached a rate comparable to that of *S. cerevisiae* and *L. thermotolerans* earlier in the process. We performed an exponential decay fit to characterize the biphasic shift of *T. delbrueckii* after 240 min (see Supplementary Figure A1). The data show that *T. delbrueckii* display a comparatively high decay constant after the biphasic shift (*b* = 0.0119 min^−1^) compared to the initial decay constants of *S. cerevisiae* and *L. thermotolerans*. This reflects the shift in predation from an exhausted prey species to the more abundant prey. Taken together, the species-specific prey decline contrasts define three distinct kinetic regimes.

The control condition for the consortia without *S. schoenii* (Fig. 1b) shows that *S. cerevisiae* has a longer lag phase compared to *L. thermotolerans and T. delbrueckii* (Shekhawat et al. 2017). The lower rate of predation of *T. delbrueckii* in Fig. 1a could be attributed to its comparatively high growth rate in the aerobic assay conditions, but this conflicts with the data observed for *L. thermotolerans*.

In single-species, co-culture predation assays, a similar decline trend could be detected (Fig. 1c). *Lachancea thermotolerans* declined the fastest, followed by *S. cerevisiae*, with *T. delbrueckii* persisting the most. A difference between the monocultures and the co-cultures is that the two non-*Saccharomyces* species showed a shorter lag-phase than *S. cerevisiae* in our experimental conditions, and growth appeared to compensate for predation pressure to some degree.

**Figure 2.**
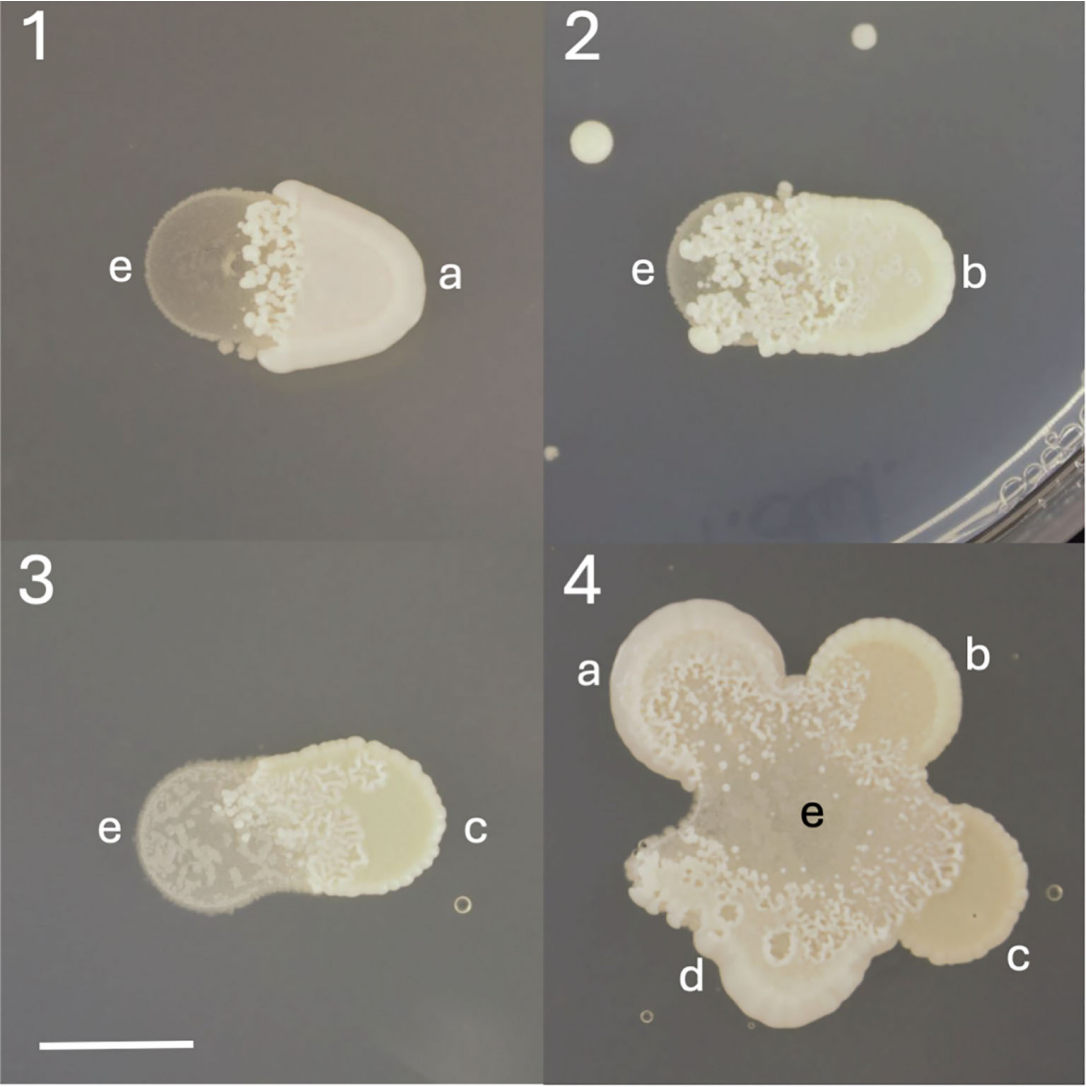
Predation screens of *S. schoenii* spotted next to prey species on YNB agar supplemented with CSM-Met after 5 days. Lobate morphology indicates predation of prey species. (1) *Saccharomycopsis schoenii* (e) spotted with *S. cerevisiae* VIN13 (a). (2) *Saccharomycopsis schoenii* (e) spotted with *T. delbrueckii* (b). (3) *Saccharomycopsis schoenii* (e) spotted with *L. thermotolerans* (c). (4) *Saccharomycopsis schoenii* (e) spotted together with *S. cerevisiae* (a), *T. delbrueckii* (b), *L. thermotolerans* (c), and *S. cerevisiae* BM45 (d). Scale bar indicate 10 mm.

In addition to the consortia, we also assessed predatory behaviour on agar plates. In Fig. 2, yeast strains and species were evaluated visually for predation after 5 days. The interface between *S. schoenii* and prey yeast shows a distinct lobate morphology. Predation is apparent across all species, but the plate assays did not allow for a quantitative comparative analysis of predation.

Taken together, the data show that all species evaluated were preyed upon efficiently by *S. schoenii*, but that some species appeared to be preferred over others. *Saccharomyces cerevisiae* was preyed upon with high efficiency, justifying the use of this species as a model to evaluate prey cell properties and responses to predation. All work from here on was carried out using *S. cerevisiae* strains to evaluate the impact of specific properties of prey cells on predation efficiency.

**Figure 3.**
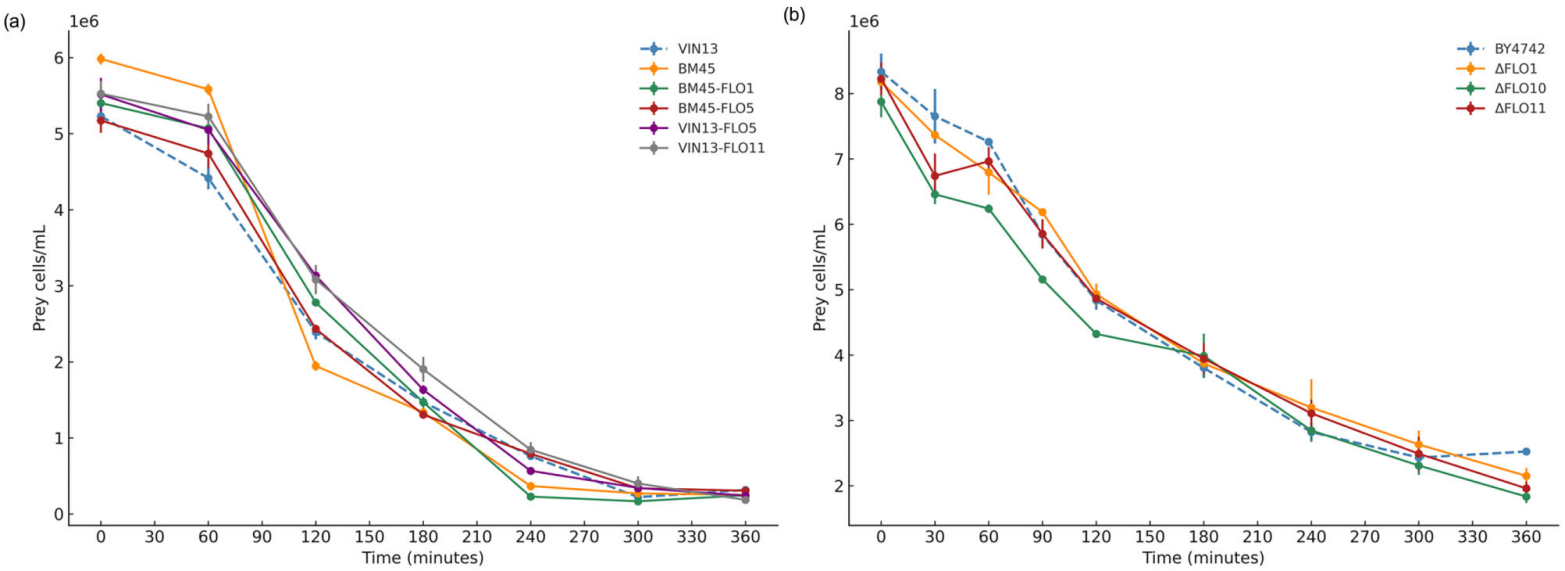
Line graphs representing the mean prey cell density and decline for prey species in co-culture with *S. schoenii*. The plots show the decline in viable cell density of prey yeast over a 360-min assay period. (a) *Saccharomyces cerevisiae* VIN13 (blue, dashed line) represents the model strain. (b) *Saccharomyces cerevisiae* BY4742 (blue, dashed line) represents the control condition for the FLO-deletion mutants screens.

To assess whether prey adhesion properties would impact predation efficiency, we screened multiple *S. cerevisiae* BM45 and VIN13 FLO-overexpressing mutants (Govender et al. 2010), and *S. cerevisiae* BY4742 FLO-deletion mutants against *S. schoenii* to investigate the impact of prey adhesion phenotypes on predation efficiency. The data in Fig. 3a show that FLO-overexpression mutants of *S. cerevisiae* BM45- and VIN13 strains do not alter the predation efficiency. A similar observation was made when *S. schoenii* was co-cultured with *S. cerevisiae* BY4742 and FLO-deletion mutants, with all prey declining at similar rates (Fig. 3b). There were no meaningful differences observed in prey dynamics and predation efficiency.

### Quantitative analysis of *S. cerevisiae* VIN13 cell wall mutants

**Figure 4.**
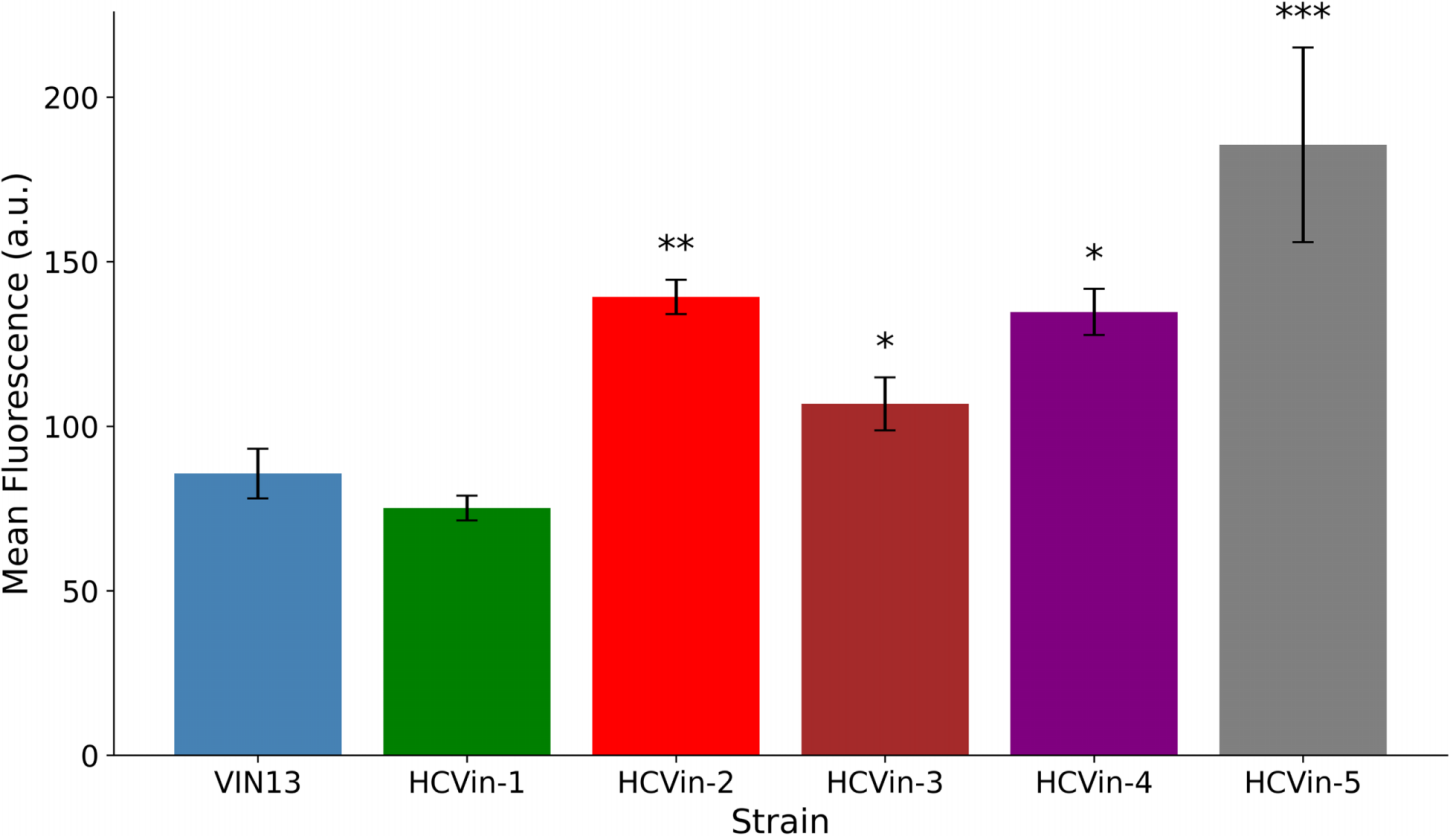
Quantification of calcofluor white fluorescence of *S. cerevisiae* VIN13 cell wall mutant strains with *S. cerevisiae* VIN13 as the control. The bar plot shows the mean calcofluor white fluorescence intensity. Fluorescence values represent the mean chitin-bound calcofluor white fluorescence. Data are presented as the mean ± SEM across all biological replicates. *P < 0.05, ** *P <* 0.01, *** *P <* 0.001.

To assess how cell wall characteristics influenced predation efficiency, we quantified chitin-associated calcofluor white fluorescence of six VIN13-derived mutant strains (named HCVin-1 to -5) that had been isolated for increased cell wall chitin levels as described in Chuene et al. (2024). Specific chitin levels were measured using quantitative confocal microscopy on five independent biological replicates of each mutant strain. Figure 4 shows the mean calcofluor white fluorescence intensity of these strains and of the VIN13 WT strain. HCVin-5 showed the highest fluorescence intensity. HCVin-4 and HCVin-2 showed intermediate fluorescence intensity, while HCVin-1 revealed a WT-like fluorescence. Dunnett’s one-sided test confirmed that HCVin-2, HCVin-4 and HCVin-5 showed significantly higher mean fluorescence intensity compared to the VIN13 strain (see Supplementary Table A1).

### Evaluating the predation efficiency of *S. schoenii* against *S. cerevisiae* VIN13 cell wall mutants

**Figure 5.**
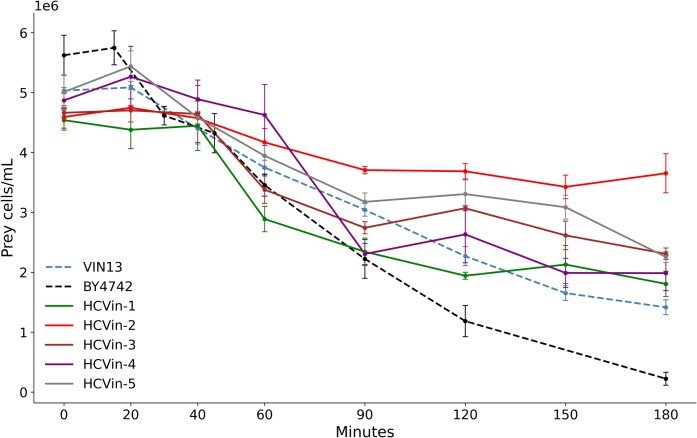
A representation of the mean prey cell density and decline for each VIN13 mutant. Each curve shows the decline in viable cell density over a 180-min assay period, reflecting trends of differential susceptibility of cell wall mutants compared to the VIN13 control. *S. cerevisiae* BY4742 is shown for reference.

Figure 5 shows the prey pairings with *S. schoenii* and *S. cerevisiae* VIN13 high chitin mutants and VIN13-WT and *S. cerevisiae* BY4742 as the control. Over the experimental time course, VIN13 WT displayed a relatively linear pattern of decline as observed previously. The data show a delay in initial prey decline at timepoints 0–40 min, followed by a steep decline in prey for all strains except HCVin-2. *Saccharomyces cerevisiae* BY4742 shows the most drastic decline in cell density over the time course. HCVin-1, HCVin-4, and VIN13 show the highest rate of cell decline after 40 min. HCVin-3 and HCVin-5 declined more gradually and less pronouncedly compared to VIN13. Conversely, HCVin-2 showed the least decline over the experimental period, with cells declining by less than half of the initial inoculation density.

### Mortality dynamics and predation efficiency of chitin mutants

**Figure 6.**
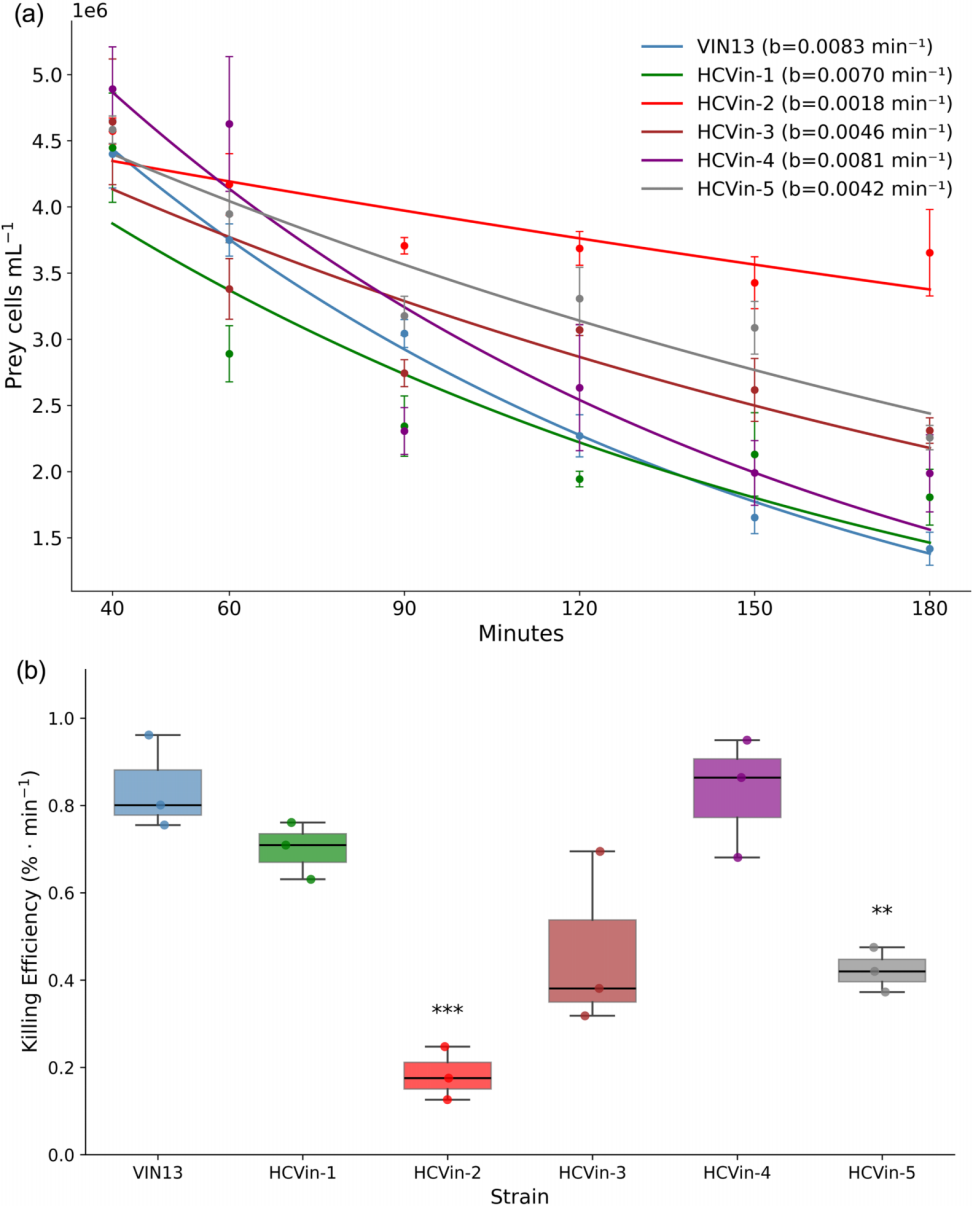
(a) Exponential decay fit of prey yeast survival under predation by *S. schoenii*. Lines represent fitted exponential decay curves, while points show mean observed survival (±SE) from three biological replicates. The figure legend displays the constant fractional loss rate per minute as a fraction of a percentage. (b) The killing efficiency of *S. schoenii* against prey mutants during predation. Boxes represent the interquartile range (IQR) with medians indicated. Whiskers extend to 1.5 × IQR. VIN13 is shown as the control condition. Lower values correspond to reduced killing efficiency, indicating slower prey decline. *P < 0.05, ** *P <* 0.01, *** *P <* 0.001.

Prey dynamics were modelled to an exponential decay fit (EDF) model, excluding the first two time points representing the adhesion phase. This allowed quantitative comparison of prey susceptibility and decline rates between mutant and WT strains. No clear trends were observed between calcofluor white fluorescence and decay constants (*b*) (Fig. 6a), as mutants expressing WT-like and high calcofluor white fluorescence, HCVin-3 and HCVin-5, declined more slowly. HCVin-2 stood out from its counterparts as the most resistant, with the lowest decay constant among all strains tested in this study.

Killing efficiency was calculated using the EDF model and fitted separately to each biological replicate of each prey mutant (Fig. 6b; Supplementary Table A2). Dunnett’s one-sided test confirmed that the killing efficiency of *S. schoenii* varied among all mutants compared to the WT strain. HCVin-1 and HCVin-4 displayed trends within a similar range to VIN13 and did not differ significantly in the Dunnett test (see Supplementary Table A3). However, HCVin-2 and HCVin-5 showed significant reductions in killing efficiency relative to VIN13. HCVin-2 was the most prominent outlier. HCVin-3 and HCVin-5 demonstrated an intermediate level of resistance.

**Figure 7.**
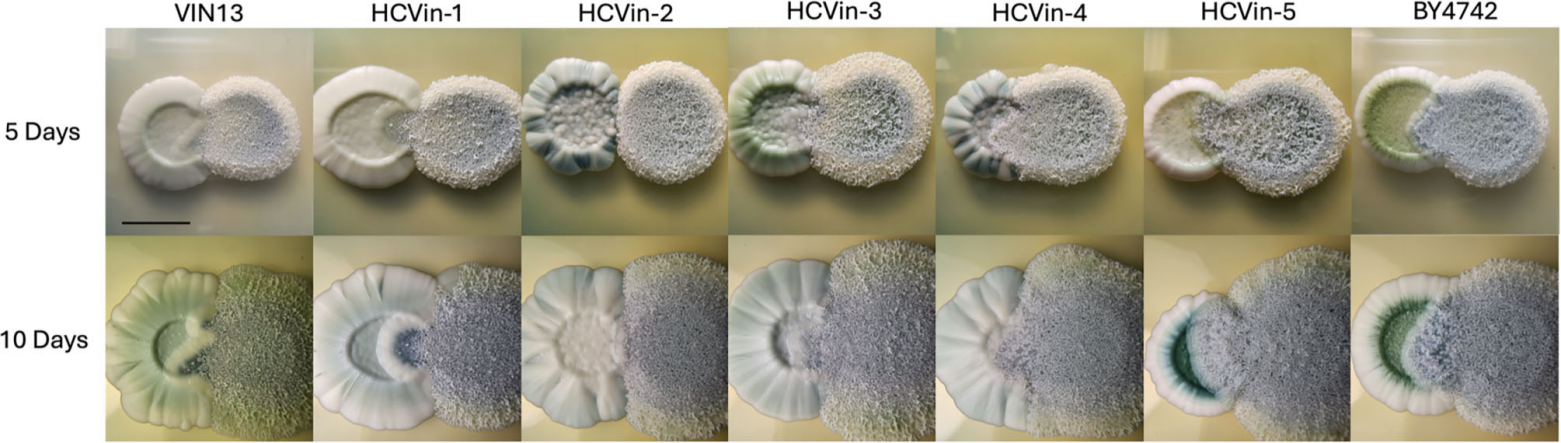
Predation screens of *S. schoenii* (right colony on each picture) spotted next to prey species (left colony on each picture) on WL agar after 5 and 10 days. Pictures show trends of differential susceptibility of mutants compared to the VIN13 control and *S. cerevisiae* BY4742.

We assessed the predatory behaviour of *S. schoenii* on WL agar plates as a qualitative complement to the liquid assays. Figure 7 shows differential colony interactions, with HCVin-2 maintaining a clear boundary with *S. schoenii* at day 10, when other prey colonies appear to have been invaded. However, it must be noted that colony morphology on plates may be influenced by both predation efficiency and growth rate differences among mutant strains, limiting the interpretive power of this assay. Despite this, the phenotype of HCVin-2 and BY4742 aligns with the pronounced resistance observed in quantitative liquid assays (Figs 5 and 6) and supports its usefulness as a rapid screening tool for identifying predation-resistant candidates.

**Figure 8.**
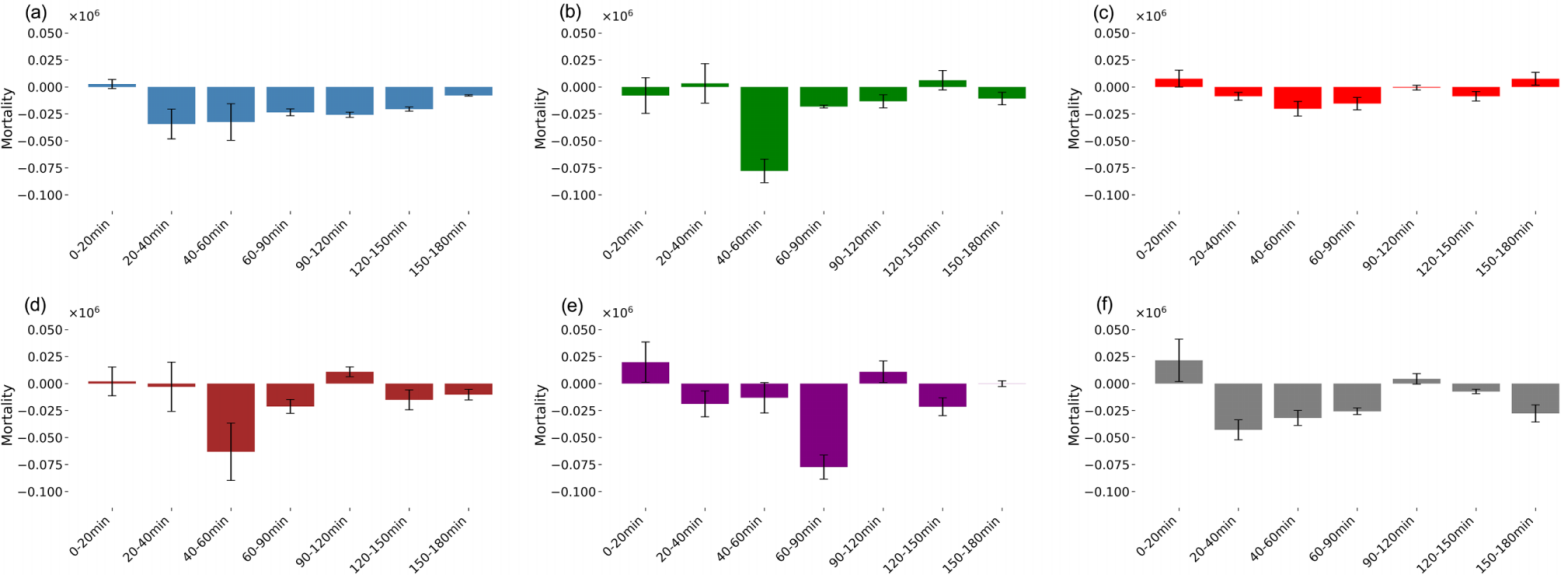
The mean mortality rates for all mutant strains. Plots show the mean mortality rate (Δcells/min) (±SE) of prey mutants. The *x*-axis represents time points in minutes. (a) *Saccharomyces cerevisiae* VIN13 WT (blue); (b) HCVin-1 (green); (c) HCVin-2 (red); (d) HCVin-2 (brown); (e) HCVin-4 (purple); and (f) HCVin-5 (grey). The *y*-axis shows the Mortality rate (Δcells/min). Negative mortality values indicate predation, while positive values represent growth.

Mean killing rates were calculated to visualize the mortality rate of mutants over time. Any divergence in kill rate kinetics observed in mutant strains would indicate a difference in predatory efficiency compared to VIN13 WT. Figure 8 shows that predation kinetics are different among the chitin mutant strains. Furthermore, it suggests again that there is no clear link between mortality rate and chitin quantification. VIN13 WT shows a steady decline, whereas the mutants have a sudden onset after 40 min. This may relate to more time required by *S. schoenii* to digest cell walls, but the same number of cells are attacked at the onset of predation. The gradual decline in kill rate across all mutants suggests an aggressive initial killing interaction followed by a decline as prey become less abundant.

## Discussion

This study advances our understanding of prey preferences and resistance phenotypes within a tractable wine fermentation ecosystem. Using *S. schoenii* as a model predator for its superior molecular and predatory characterisation, we evaluated predation dynamics across several wine-associated yeasts and examined the role of prey adhesion phenotypes and cell wall components. This research provides new insights into the specificity and kinetics of predatory yeast interactions.

The results show clear, species-specific differences in predation dynamics in a consortium. *Lachancea thermotolerans* exhibited a heightened, early susceptibility and steep decline, while *S. cerevisiae* showed a steady, more linear decline. *Torulaspora delbrueckii* displayed a biphasic trajectory with increasing susceptibility only after other prey declined drastically. This sequential predation pattern may mirror ecological 'prey switching' behaviour, where predators opportunistically target the most abundant prey (Murdoch 1969). However, it is important to note that underlying cues such as cell wall traits, metabolite secretion and stress-induced vulnerability among prey must first be clarified. These observations represent density-dependent opportunism, which is common among microbial predators (Bohannan and Lenski 2000, Hiltunen and Becks 2014).

The FLO-mediated variation in prey cell adhesion had no discernible impact on predation. This emphasizes that Flo-adhesins likely do not aid nor hinder prey attachment and resulting penetration. In contrast, differences in cell wall chitin content, quantified by chitin-bound calcofluor white fluorescence, were correlated with, but did not dictate, the degree of resistance of prey mutant strains of *S. cerevisiae* VIN13. Four of the VIN13-related chitin mutants showed reduced predation efficiency. While this strongly suggests a link between chitin levels and predation tolerance, this link is not linear. Interestingly, this aligns well with the data of Chuene et al. (2024), which showed that while chitinase binding to cell wall chitin was correlated with cell wall chitin levels, this correlation was not linear. This suggests that other parameters, particularly the exact distribution of chitin and the structural integrity of the cell walls in these mutants, play a significant role (Lipke and Ovalle 1998, Klis et al. 2002).

A key finding in this study is the identification of HCVin-2 and HCVin-5, which demonstrated pronounced resistance phenotypes. These phenotypes, presented through reduced decay constants and significantly lower killing efficiencies, suggest specific genetic changes fundamental to resistance. The lack of a simple correlation between chitin content and survival prompts several mechanistic hypotheses. First, prey resistance may require complex restructuring or cross-linking of prey cell wall polymers (Kollár et al. 1997, Klis et al. 2006, Cabib et al. 2007). Second, adaptive cell wall remodelling or stress responses, rather than static biochemical composition, could determine susceptibility (Levin 2005, Bermejo et al. 2008, Sanz et al. 2018). And finally, noncell wall factors, including prey’s metabolic state, signal transduction, or efflux systems, might contribute to prey survival under predatory attack (Schuller et al. 1994, Piper et al. 1998, Hohmann 2002).

However, a critical limitation in our understanding of *Saccharomycopsis’* ecological interactions is the absence of direct observational evidence for its interactions with evolutionarily relevant prey in natural environments. Notably, isolation studies have reported previously that they recovered nearly half of all *S. schoenii* isolates from oak exudates distributed across the Northern Hemisphere (Kurtzman and Smith 2011). Nevertheless, despite reports of *Saccharomycopsis* species ranging from diverse and often managed habitats, systematic amplicon or shotgun sequencing studies of soil, vineyard, and plant-associated microbiomes have not revealed significant populations of *S. schoenii* (Kowallik et al. 2015, Santos et al. 2023, Dost et al. 2024, Hu et al. 2025). This likely reflects both technical limitations, e.g. sequencing biases or detection sensitivity, and ecological realities whereby both predator and its natural, evolutionarily relevant prey are genuinely rare or sporadically distributed in nature (Goddard et al. 2010, Bokulich and Mills 2013, Pinto et al. 2014, Kowallik et al. 2015). Our inability to sample natural predation events or ecological networks limits our ability to generalize laboratory predator–prey outcomes to real ecosystems, emphasising a significant gap between experimental tractability and ecological realism.

Therefore, our results, while insightful, are limited to experimental systems and may not directly reflect interactions in wild or fermentation contexts. *Saccharomyces cerevisiae* can provide a powerful platform for dissecting relevant prey traits, but the ecological relevance of these predatory interactions and their impact in natural ecosystems remains untested.

Overall, this study provides a mechanistic framework for dissecting *bona fide* predatory interactions between *S. schoenii* and prey yeast. It identified new candidate resistance strategies and highlights the disconnect between standardized laboratory models and true ecological observation. Only by integrating genetic, physiological, and ecological approaches can we fully resolve the evolutionary drivers and potential impact of predatory yeast behaviours in complex microbial communities.

## Supplementary Material

foaf075_Supplemental_Files
