# Supplementary material for: Prey preference and cell wall-mediated resistance shape predation efficiency in Saccharomycopsis schoenii
Source: FEMS Yeast Res. 2026 Jan 2;26:foaf075. doi: 10.1093/femsyr/foaf075 (PMC12857228; doi:10.1093/femsyr/foaf075)
Supplement: foaf075_Supplemental_Files [file foaf075_supplemental_files.zip › Supplementary Table A1.docx]

**Supplementary Table A1:** Dunnett’s one-sided test for mutant strains exhibiting significantly higher chitin fluorescence intensity than VIN13. The table reports the mean differences, standard errors, test statistics, and multiplicity-adjusted p-values for this directional hypothesis. Mutants with adjusted p-values below 0.05 can be interpreted as having significantly greater chitin content relative to VIN13 under the one-sided test.

| Strain | Dunnett estimate^a^ | SE^b^ | t-value^c^ | adj. p-value^d^ | Significance^e^ |
| --- | --- | --- | --- | --- | --- |
| HCVin-1 | -10,453 | 20,204 | -0,517 | 0,5194 | ns |
| HCVin-2 | 53,668 | 20,204 | 2,656 | 0,0307 | * |
| HCVin-3 | 21,199 | 20,204 | 1,049 | 0,3748 | ns |
| HCVin-4 | 49,089 | 20,204 | 2,43 | 0,0497 | * |
| HCVin-5 | 99,888 | 20,204 | 4,944 | >0,0000 | *** |

^a^ Dunnett estimate - positive strain mean > VIN13 WT

^b^ Standard error

^c^ Test statistic

^d^ Multiplicity-adjusted p-values for the one-sided directional hypothesis

^e^ Significance: *p < 0.05; ** p < 0.01, *** p < 0.001; ns = not significant
