# Supplementary material for: Prey preference and cell wall-mediated resistance shape predation efficiency in Saccharomycopsis schoenii
Source: FEMS Yeast Res. 2026 Jan 2;26:foaf075. doi: 10.1093/femsyr/foaf075 (PMC12857228; doi:10.1093/femsyr/foaf075)
Supplement: foaf075_Supplemental_Files [file foaf075_supplemental_files.zip › Supplementary Table A2.docx]

**Supplementary Table A2**: Replicate-level exponential decay fits of prey survival under predation by S. schoenii

| Strain | Replicate | $\boldsymbol{a}$^a^ | *b*^b^ | Killing Efficiency^c^ |
| --- | --- | --- | --- | --- |
| VIN13 | 1 | 4,69E+06 | 0,00801 | 0,80096 |
| VIN13 | 2 | 4,38E+06 | 0,00961 | 0,96128 |
| VIN13 | 3 | 4,25E+06 | 0,00755 | 0,75520 |
| HCVin-1 | 1 | 3,90E+06 | 0,00631 | 0,63073 |
| HCVin-1 | 2 | 4,41E+06 | 0,00709 | 0,70914 |
| HCVin-1 | 3 | 3,32E+06 | 0,00761 | 0,76092 |
| HCVin-2 | 1 | 4,29E+06 | 0,00175 | 0,17538 |
| HCVin-2 | 2 | 4,21E+06 | 0,00248 | 0,24752 |
| HCVin-2 | 3 | 4,54E+06 | 0,00126 | 0,12606 |
| HCVin-3 | 1 | 3,94E+06 | 0,00318 | 0,31807 |
| HCVin-3 | 2 | 4,77E+06 | 0,00695 | 0,69470 |
| HCVin-3 | 3 | 3,75E+06 | 0,00381 | 0,38092 |
| HCVin-4 | 1 | 5,55E+06 | 0,00681 | 0,68091 |
| HCVin-4 | 2 | 4,66E+06 | 0,00949 | 0,94947 |
| HCVin-4 | 3 | 4,41E+06 | 0,00864 | 0,86392 |
| HCVin-5 | 1 | 4,75E+06 | 0,00420 | 0,41991 |
| HCVin-5 | 2 | 4,20E+06 | 0,00373 | 0,37267 |
| HCVin-5 | 3 | 4,25E+06 | 0,00475 | 0,47483 |

^a^ Initial quantity of prey cells mL^-1^.

^b^ Exponential decay constant (min^-1^).

^c^ Killing efficiency of *S. schoenii* against prey as a change in fraction of the prey population over time (% min^-1^).
