# Supplementary material for: Prey preference and cell wall-mediated resistance shape predation efficiency in Saccharomycopsis schoenii
Source: FEMS Yeast Res. 2026 Jan 2;26:foaf075. doi: 10.1093/femsyr/foaf075 (PMC12857228; doi:10.1093/femsyr/foaf075)
Supplement: foaf075_Supplemental_Files [file foaf075_supplemental_files.zip › Supplementary Table A3.docx]

**Supplementary Table A3:** Statistical comparisons performed using one-sided Dunnett’s tests (cell wall mutants < VIN13) against the killing efficiency of S. schoenii during predation of S. cerevisiae VIN13 and cell wall mutant strains.

| Strain | Dunnett estimate | SE^a^ | t-value^b^ | adj. p-value^c^ | Significance^d^ |
| --- | --- | --- | --- | --- | --- |
| HCVin-1 | -0,1389 | 0,0895 | -1,5524 | 0,4660 | ns |
| HCVin-2 | -0,6562 | 0,0879 | -7,4675 | >0,0000 | *** |
| HCVin-3 | -0,3746 | 0,1619 | -2,3137 | 0,1460 | ns |
| HCVin-4 | -0,0077 | 0,1236 | -0,0624 | 1,0000 | ns |
| HCVin-5 | -0,4167 | 0,0846 | -4,9235 | 0,0011 | ** |

^a^ Standard error

^b^ Test statistic

^c^ Multiplicity-adjusted p-values for the one-sided directional hypothesis

^d^ Significance: *p < 0.05; ** p < 0.01, *** p < 0.001; ns = not significant
