# Supplementary figures and images for: Prey preference and cell wall-mediated resistance shape predation efficiency in Saccharomycopsis schoenii
Source: FEMS Yeast Res. 2026 Jan 2;26:foaf075. doi: 10.1093/femsyr/foaf075 (PMC12857228; doi:10.1093/femsyr/foaf075)

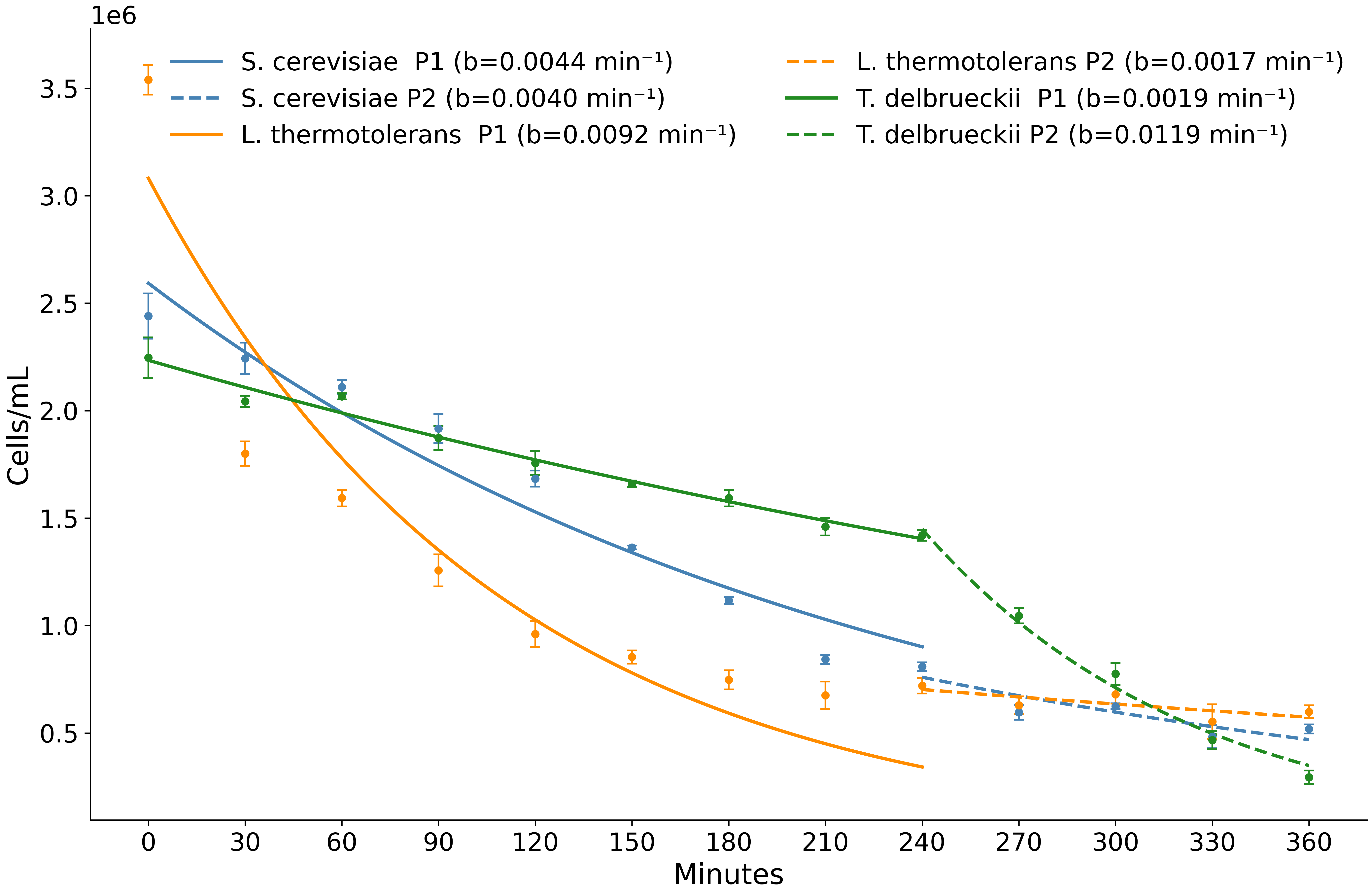

Supplement: foaf075_Supplemental_Files [file foaf075_supplemental_files.zip › Supplementary Figure A1.tiff]
